# Neurodegeneration-related genes influence *C. elegans* pharyngeal activity

**DOI:** 10.17912/micropub.biology.000897

**Published:** 2024-03-13

**Authors:** Hannah Selvarathinam, Aladin Elkhalil, Walter E. Schargel, Piya Ghose

**Affiliations:** 1 Department of Biology, The University of Texas at Arlington, Arlington, Texas, United States

## Abstract

Pharyngeal pumping and its reduction following mechanical insult are well-studied *C. elegans* behaviors. Here, we assessed new applications of pharyngeal pumping assays in the study of neurodegenerative disease and psychiatric illness. We examined five genes implicated in two forms of neurodegeneration, Hereditary Spastic Paraplegia (HSPs) and Alzheimer’s Disease (AD), for both baseline pharyngeal pumping and the depressive response after touch stimulus. All five mutants showed reduced baseline pumping rate, suggesting a potential utility of this assay to study neurodegenerative disease on a broad scale. However, regarding the induced pumping response, which has been linked to schizophrenia, only specific genes, the HSP-related *atln-1/* Atlastin and the AD-related *ptl-1/* tau, showed defects. Together, we highlight two pharyngeal pumping behaviors as genetically distinct, potentially informative settings for understanding the functions of genes linked to neurodegeneration.

**Figure 1.**
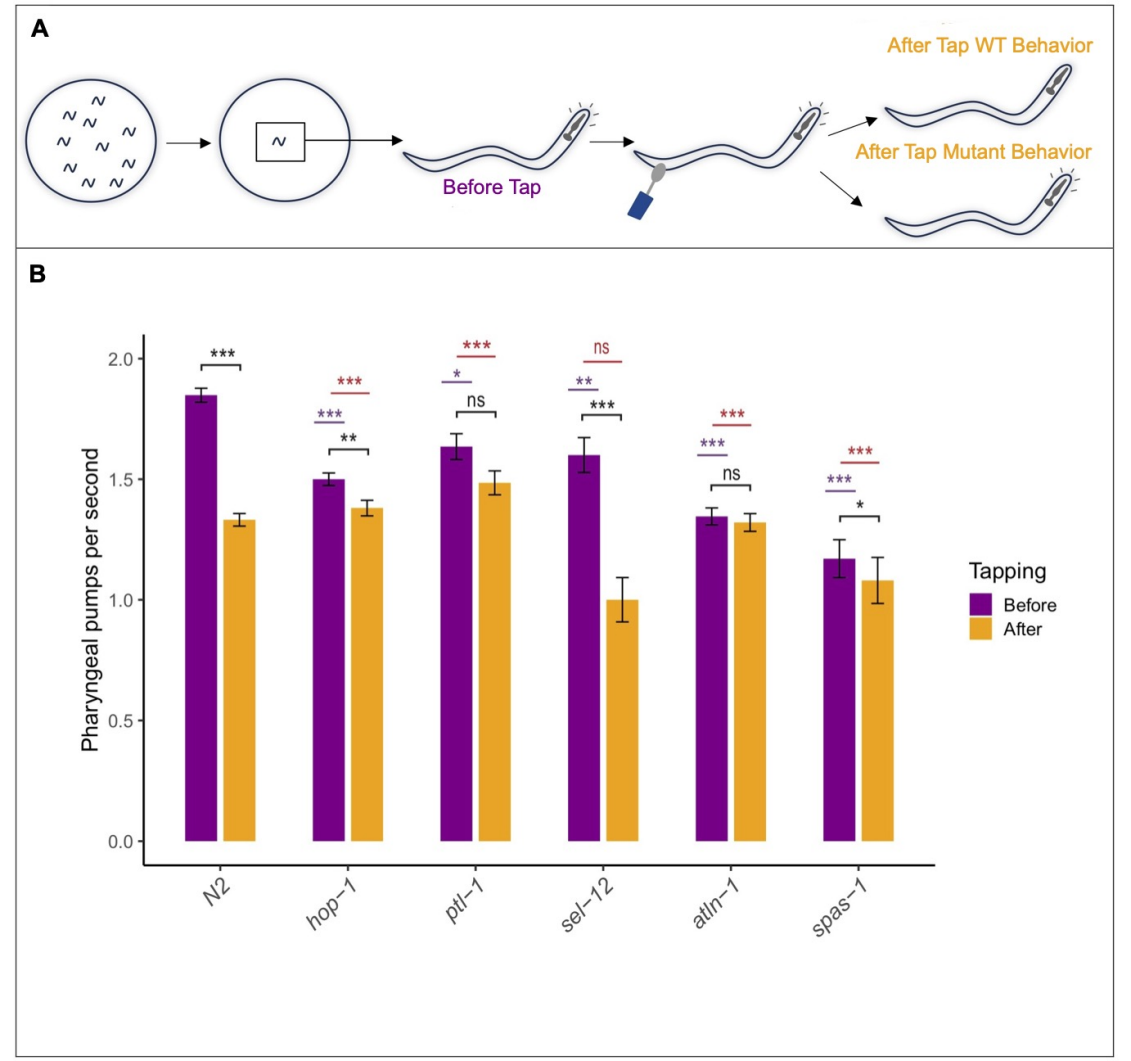
**(A)** Schematic of experimental paradigm. **(B)** Quantification of pharyngeal pumps per second. Purple bar, before tap; orange bar, after tap. Black lines, paired t-test (Before and after compared within genes) with Bonferroni correction; purple lines, Dunnett's test comparing pharyngeal pumping at base against N2 from two-factor ANOVA; red lines, Dunnett's test comparing the difference in pharyngeal pumping response (Before - After) from two-factor ANOVA. Asterisks convention: *** for p<0.001, ** p<0.01, * p< 0.05.

## Description

Pharyngeal pumping of the feeding organ is a stereotyped feeding behavior in the nematode *C. elegans* (Albertson and Thomson 1976) regulated by 4 of the 20 neurons of the pharyngeal nervous system and driven by pharyngeal muscles. Acetylcholine synthesis and packaging, a nicotinic acetylcholine receptor, GABA and dopamine have also been reported to be important for pharyngeal pumping (Avery and Horvitz 1990; Dwyer 2018) . Following touch stimulus (tail tap) in wild-type worms, pharyngeal pumping rate is inhibited, a genetically encoded response involving the innexin and glutamatergic pathways (Keane and Avery 2003) . Interestingly, this depression in pumping rate following mechanical stimulation has been linked to both neurodegeneration (Stravalaci et al. 2012) and psychiatric disease (Dwyer 2018) .

The reduced pumping rate has been compared to a "startle response/reflex" (Dwyer 2018) , a fast motor response to a sudden stimulus, such as a loud noise (Fetcho and McLean 2009) . Disruptions in this response are linked to neuropsychiatric diseases (Hallet 2012) , such as schizophrenia. In *C. elegans* , the "startle response" defect can be modulated by known psychotropic drugs and has been suggested as an assay for schizophrenia-like behavior (Dwyer 2018) .

Pharyngeal pumping rate reduction has also been linked to Alzheimer’s Disease (AD), a common form of dementia (Knopman et al. 2021, Eimer, Donhauser, and Baumeister 2002) . Pumping rate has been shown to be reduced following exposure to the AD-related Aβ _1–42_ oligomer (Stravalaci et al. 2012) which also causes neuronal dysfunction, neurodegeneration as well as physiological defects (Gallrein et al. 2021) .

The diagnoses of neurodegenerative disease can be an invasive, delayed and expensive process (Manera et al. 2023) . As such, increased efforts are being made to develop alternative means of pre-assessment for a potential neurodegenerative condition to then be tested in a more specialized manner. Moreover, some neurodegenerative and psychiatric diseases have been shown to have common epidemiological links (Wingo et al. 2022) and there is much interest in better understanding pathological links as well. *C. elegans* has proven to be an increasingly informative model to understand the fundamentals of neurodegenerative disease (Caldwell et al. 2020) . In this study, we explored the utility of the *C. elegans* pharyngeal pumping behavior in the study of neurodegenerative disease and possible links with psychiatric disease.

We performed a pilot candidate gene screen (Selvarathinam 2022) for tail-tap-induced inhibition of pharyngeal pumping rate ( **FIG 1A)** across mutants for 5 genes with mammalian homologs linked to two forms of neurodegeneration, Hereditary Spastic Paraplegia (HSPs), a family of inherited neurodegenerative diseases characterized by progressive weakness and stiffness of the legs (Shribman et al. 2019; Evans et al. 2006) and AD. Among the HSP genes, we examined mutants for *spas-1* /Spastin, which encodes a microtubule-severing ATPase ( **FIG 1B** ) and *atln-1* /Atlastin, which encodes a dynamin-like protein important for endoplasmic reticulum (ER) network stabilization. We also considered three AD-related genes in our screen. We looked at mutants for two genes encoding homologs of the hydrolytic g-secretase component presenilin (Knopman et al. 2021; Li and Greenwald 1997; Eimer, Donhauser, and Baumeister 2002) , *hop-1* and *sel-12* . We also examined *ptl-1* /tau mutants. The gene *ptl-1* encodes a homolog of tau (Barbier et al. 2019) , a microtubule-associated protein that stabilizes microtubules (Barbier et al. 2019, Medeiros, Baglietto-Vargas, and LaFerla 2011) .

Briefly, in our screen, we counted N2 wild-type and mutant worm pharyngeal pumping over 20 seconds under two conditions, prior to tail tapping and directly afterwards. For every trial conducted with a given genotype, N2 wild-type controls were also assayed. We then proceeded to analyze our counts according to three parameters: (1) base-line pumping rate across genotypes compared to N2 wild-type, (2) before and after tap differences in pharyngeal pumping for each mutant genotype, and (3) the differences in the pharyngeal pumping rates before and after tap of each mutant compared to this difference in wild-type.

First, and interestingly, we found that all 5 mutants tested had a significantly lower pharyngeal basal pumping rate ( **FIG 1B** , before tail tap, purple bars, purple asterisk) compared to wild-type N2 worms. These data suggest a potential application of baseline pharyngeal pumping in the study of different forms of neurodegeneration at the behavioral level and possibly a more fundamental role for microtubule dynamics, given defects seen in both *spas-1* /Spastin and *ptl-1* /Tau. We also propose the idea that baseline pharyngeal pumping rate may be considered for use as a potential assay for the study of the genetics behind HSPs, perhaps as a readout of spasticity.

We then sought to quantify and analyze the before and after tapping pharyngeal “startle” response with the goal of assessing the relevance of this induced behavior to the study of neurodegenerative disease and for finding links with schizophrenic behavior. First, we compared the before and after tap response ( **FIG 1B** ), black lines and black asterisk) for each individual genotype. As expected, wild-type worms showed a significant reduction in pharyngeal pumping after tapping ( **FIG 1B** ), black lines, black asterisk). Interestingly, of our tested mutants, only two, one from each disease group, *atln-1 /* Atlastin and *ptl-1 /* Tau, lacked this reduction in pumping rates ( **FIG 1B** ), thereby having a ‘startle’ response defect. The pumping rates of both these mutants before and after tap were not significantly different ( **FIG 1B** ), and in line with this, the difference in pumping rates were significantly different from this difference in wild-type ( **FIG 1B** , red lines and red asterisk). Conversely, we found that the AD-linked *sel-12 /* presenilin mutants behaved as wild-type both in trend and degree of pharyngeal pumping response - there was a significant difference pre and post tap ( **FIG 1B** ) but no difference in the pumping frequency difference from wild-type ( **FIG 1B** ). Interestingly, while the AD-related *hop-1 /* presenilin and HSP-related *spas-1 /* Spastin mutants showed reduced pumping rates after tap, as in wild-type, the difference in the pumping rates before and after tap compared to this difference in wild-type was significantly different from wild-type. The ‘startle response’ of *sel-12 /* presenilin mutants specifically was as large as wild-type, even though the basal pumping rate was smaller; whereas for all other mutants both their basal pumping rate and ‘startle response’ rate were smaller than compared to wild-type. This suggests that the reduction in pumping rate trend and the degree of this reduction may have different implications. Based on these data, if the pharyngeal pumping inhibition is indeed considered a reliable measure of a “startle response’, there may be no direct genetic link between the schizophrenia-related startle, AD and HSP, or neurodegeneration on a broad scale.

Our study highlights differences in the well-studied behaviors of baseline pharyngeal pumping and pharyngeal pumping inhibition after mechanical stimulus with respect to the role of genes related to neurodegeneration. We suggest new possible applications of baseline pharyngeal pumping behavior in the study of AD and of spasticity during HSPs specifically, perhaps expanding to other pathologies. Such work would allow for the further exploration of behavior as a predictive metric for neurodegenerative diseases rooted in genetic mutations. We propose the evaluation of additional panels of genes implicated in neurodegeneration for validation. Our study also underscores the genetic complexity of pharyngeal pumping inhibition with respect to genes associated with neurodegeneration and suggests that the different genes tested here may affect this behavior through their more specific functions as opposed to the roles they play in a common disease. We propose that studying the genetic differences between baseline pumping and its induced reduction may also be an interesting avenue to pursue.

Expansion of our pilot study would take into consideration several additional facets of the highly intricate pharyngeal pumping behavior at both the technical and mechanistic level. On a technical level, an advanced study would entail visual examination of movies acquired under both conditions to aid detection of subtle behaviors. The study would also be done with added precision measuring responses in the presence, absence and through the exposure-deprivation of food for an extended period (per Dalliere et al. 2016).

In addition, additional genes would be considered, together with their possible interactions with the genes tested here, which would also be tested for neuron-specific function. Based on multiple studies revealing systemic control of pumping behavior by the body wall muscle (Izquierdo et al. 2022, Takahashi et al. 2017) , additional genes would be tested. It has been demonstrated that cholinergic transmission of the body wall via the neuro-muscular junction inhibits feeding behavior in tandem with locomotion involving of the muscle receptor LEV-1 (Izquierdo et al. 2022) . In addition, optical silencing studies of the body wall muscles have also revealed their involvement via the proton pump Arch in signal strength-specific manner via gap junction and dense-core vesicles (Takahashi et al. 2017) motivating the testing of related genes.

Taken together, we highlight pharyngeal pumping behaviors as promising settings to gain insights about the functions of genes related to neurodegeneration thus further broadening the scope of *C. elegans* in the study of neurodegenerative disease.

## Methods

**Methods**

***C. elegans* culture conditions:** Nematodes were cultured on E. coli OP50 plates at 20 ^ο^ C and scored at the same temperature. Wild-type animals were the Bristol N2 subspecies.

**Pharyngeal pumping behavioral assays:** Pharyngeal pumping assays involved placing ten age-matched freely-moving worms at the L4 stage onto unseeded plates and recording the number of pharyngeal pumps (movements of the grinder in the terminal bulb) for 20 seconds under a dissecting microscope by direct visualization. Inhibition assays involved then tapping each nematode near their tail with a worm pick. Pharyngeal pumping was then recorded for another 20 seconds. For every trial conducted both the control (N2, n=10) and a mutant (n=10) were assayed. Per Keane and Avery 2003, prior to recording, larvae were picked individually to unseeded NGM plates briefly to remove most of the OP50 from the body surface. Animals were further cleaned by transferring to another unseeded plate and allowed to recover for 5 min at room temperature. This was done for all scored animals of all genotypes to ensure the behaviors observed are not due to or affected by presence of food.

**Reagents.** The following strains were tested:

N2 , Bristol

RB1127: *atln-1 ( ok1144 )*

FX683 : *spas-1 ( tm683 )*

GS2447 : *hop-1 ( ar179 )*

RB809 : *ptl-1 ( ok621 )*

AN87 : *sel-12 ( ty11 )*

**Statistical Analysis:** Statistical analyses were performed using R Core Team (2013) [ *R: A language and environment for statistical computing. R Foundation for Statistical Computing, Vienna, Austria. ISBN 3-900051-07-0, URL http://www.R-project.org/* ]. Black lines represent paired t-tests (Before and After compared within genes) with a Bonferroni correction applied; purple lines the Dunnett's test comparing pharyngeal pumping at base against N2 ; and red lines for the Dunnett's test comparing the difference in pharyngeal pumping response (Before - After) against N2. The Dunnett’s tests were conducted after finding statistically significant differences (p<0.001) for both pharyngeal pumping rate at base and pharyngeal pumping response after stimulus in two-factor ANOVAs without interaction in which the different trials were defined as a random factor. The following convention for the use of asterisks was used: *** for p<0.001, ** p<0.01, * p< 0.05.

## Extended Data

Description: Detailed Statistical Analysis. Resource Type: Dataset. DOI: 10.22002/vnk2h-25266

Description: Raw Data. Resource Type: Dataset. DOI: 10.22002/18yv9-7aw57
